# Cervical lordosis in asymptomatic individuals: a meta-analysis

**DOI:** 10.1186/s13018-018-0854-6

**Published:** 2018-06-15

**Authors:** Guang-Ming Guo, Jun Li, Qing-Xun Diao, Tai-Hang Zhu, Zhong-Xue Song, Yang-Yang Guo, Yan-Zheng Gao

**Affiliations:** 1 Department of Orthopaedics, Henan Zhoukou Union Orthopaedic Hospital, East Section, Taihao Road, Zhoukou, 466000 Henan China; 2 Department of Orthopaedics, Henan Province People’s Hospital, Zhengzhou, 450000 Henan China

**Keywords:** Cervical spine, Lordosis, Asymptomatic, Age, Gender

## Abstract

**Background:**

Cervical lordosis has important clinical and surgical implications. Cervical spine curvature is reported with considerable variability in individual studies. The aim of this study was to examine the existence and extent of cervical lordosis in asymptomatic individuals and to evaluate its relationship with age and gender.

**Methods:**

A comprehensive literature search was conducted in several electronic databases. Study selection was based on pre-determined eligibility criteria. Random effects meta-analyses were performed to estimate the proportion of asymptomatic individuals with lordosis and the effect size of cervical lordotic curvature in these individuals which followed metaregression analysis to examine the factors affecting cervical lordosis. Data from 21 studies (15,364 asymptomatic individuals, age 42.30 years [95% confidence interval 36.42, 48.18], 54.2% males) were used in the present study.

**Results:**

In this population, 63.99% [95% confidence interval 44.94, 83.03] individuals possessed lordotic curvature. Degree of lordotic curvature differed by method of measurement; 12.71° [6.59, 18.84] with Cobb C2–C7 method and 18.55° [14.48, 22.63] with posterior tangent method. Lordotic curvature was not significantly different between symptomatic and asymptomatic individuals but was significantly higher in males in comparison with females. Age was not significantly associated with lordotic cervical curvature.

**Conclusion:**

Majority of the asymptomatic individuals possesses lordotic cervical curvature which is higher in males than in females but have no relationship with age or symptoms.

## Background

Cervical lordosis is important for the efficiency of many processes including mastication, breathing, vocalization, eye movement, and gaze and for the shock absorption during walking and running [1]. Curvature of the cervical spine has important clinical implications [2, 3]. Attainment of moderate cervical lordotic curvature is found to be associated with better surgical outcomes in patients with neurologic deficits [4–6]. Reattainment of cervical lordosis after a surgical intervention is also considered important as compression of nervous tissue may cause injury otherwise [7].

Cervical lordotic curvature starts becoming visible at around 10 weeks of fetal development [8] formed by the posterior wedging when height of vertebrae and discs at anterior side becomes greater than posterior side [1, 9]. Cervical spine in asymptomatic individuals generally attains lordotic alignment but up to 35% of cases exhibit kyphosis [10]. Biomechanically, a lordotic configuration can resist large compressive loads [11] and minimize stress on the vertebral body endplates [12]. Cervical spine distributes the compressive load differently as compared with the rest of the spine; 36% of the compressive load is absorbed by the anterior column and 64% by the posterior facet joints [13, 14].

There are four reliable and predictive line drawing methods for the measurement of cervical lordosis on radiographs: Cobb C2–C7 method, Ishihara’s index, Harrison C2–C7 posterior tangent method, and area under the curve [15]. Cobb method [16] was proposed for the evaluation of sagittal spinal curvature which was later modified by drawing vertebral endplate lines to construct angles on sagittal radiographs and is frequently used to evaluate cervical lordosis. Ishihara index is another way to measure curvature of the spine which is achieved by summing up the spinal lines connecting the posteroinferior corners of vertebra bodies and to construct additional orthogonal lines [17]. Harrison et al. [18] proposed a geometrical model for the measurement of cervical curvatures in sagittal radiographs which was later modified to an elliptical form to be usable for cervical lordosis as well [19]. There is a wide variation in the cervical lordotic curvature in asymptomatic individuals and patients with related conditions [9, 20, 21]. In asymptomatic individuals, average cervical lordosis is reported variably, e.g., 21.3 by Gore et al. [9], 22.3 by Owens et al. [22], and 34° by Harrison et al. [18], depending also on the method used to measure the curvature.

Increase in lordosis with age is also reported by some studies [9, 23] but not all. Moreover, whereas some studies have found that a non-lordotic sagittal cervical curvature is not related to patient’s initial symptoms [9], others have found that non-lordotic cervical curvature correlates with initial panic conditions [20, 21]. These varying observations necessitate having a systematic review of the relevant literature to have reliable estimates of normal lordotic cervical curvature and its affecting factors. The aim of present study was to review the studies which reported cervical curvature in healthy asymptomatic individuals after examining the radiographs with a reliable method, to synthesize the quantitative information pertaining to the cervical lordosis and its relationship with age and gender and morbidity.

## Methods

### Eligibility criteria

Studies were included if they met following criteria: study (1) included asymptomatic individuals either as sole study population or as controls to symptomatic patients to study normal measures of cervical lordosis, (2) provided values of C2–C7 lordosis angles and/or the proportion of individuals with lordotic curvature, and (3) included adult patients (above 18 years of age). Studies were excluded if (1) reported cervical lordosis measures other than global lordosis (C2–C7 angle), (2) reported segmental lordotic angles but not global lordosis, and (3) reported cervical lordosis measures without mentioning the symptomatic information of individuals/patients.

### Search and selection of studies

We searched Embase, Google Scholar, Ovid SP, and PubMed databases for relevant studies by using suitable MeSH and keywords for research papers published before November 2016. The search was not restricted to language or period of publication. The following search terms and strategies were used: (1) cervical-lordosis OR curvature OR lordotic curvature OR alignment; (2) angle OR Cobb angle OR posterior tangent OR theta OR; (3) Radiograph OR X-ray OR Roentgenograph; and (4) various combinations of (1), (2), and (3).

Two reviewers conducted initial database search and independently screened the titles and abstracts identified in the initial search. This followed the observance of the inclusion and exclusion criteria as a result of which full text of articles were identified and later retrieved. If additional data or clarification was necessary, we contacted the study authors. Any disagreement between reviewers was resolved by discussion with other coauthors.

### Data and analyses

The following information was collected from each study using a standardized form: study design and location, main inclusion/exclusion criteria, patient demographics, and study outcomes. Data were extracted by two reviewers independently. Cervical curvature measurements were considered if the study utilized either Cobb C2–7 angle (angle between the horizontal line of C2 lower endplate and the horizontal line of C7 lower endplate) or posterior tangent (angle formed by a line projected parallel to the posterior surface of C2 and a line parallel to the posterior surface of C7) method.

Statistical heterogeneity of the required data was tested with a chi-square test, and between-studies inconsistency was quantified by the *I*^2^ index. Meta-analyses were carried out by using Stata software (version 12; Stata Corporation, USA) by pooling the C2–C7 angles reported in individual studies and to generate inverse variance weighted overall effect size as well as effect sizes with regard to method used for the measurement of the lordotic cervical curvature and percentage of asymptomatic individuals with lordotic cervical curvature.

Random effects model was used for the meta-analyses keeping in view significant heterogeneity of the meta-analyzable data. Further analyses were carried out to evaluate the symptomatic and gender differences in cervical lordotic curvature by performing meta-analyses of mean differences using RevMan software (version 5.3; Cochrane collaboration). Meta-regression analyses were also performed to evaluate the effect of age and gender on cervical lordotic curvature using restricted maximum likelihood method in Stata software.

## Results

**Fig. 1:**
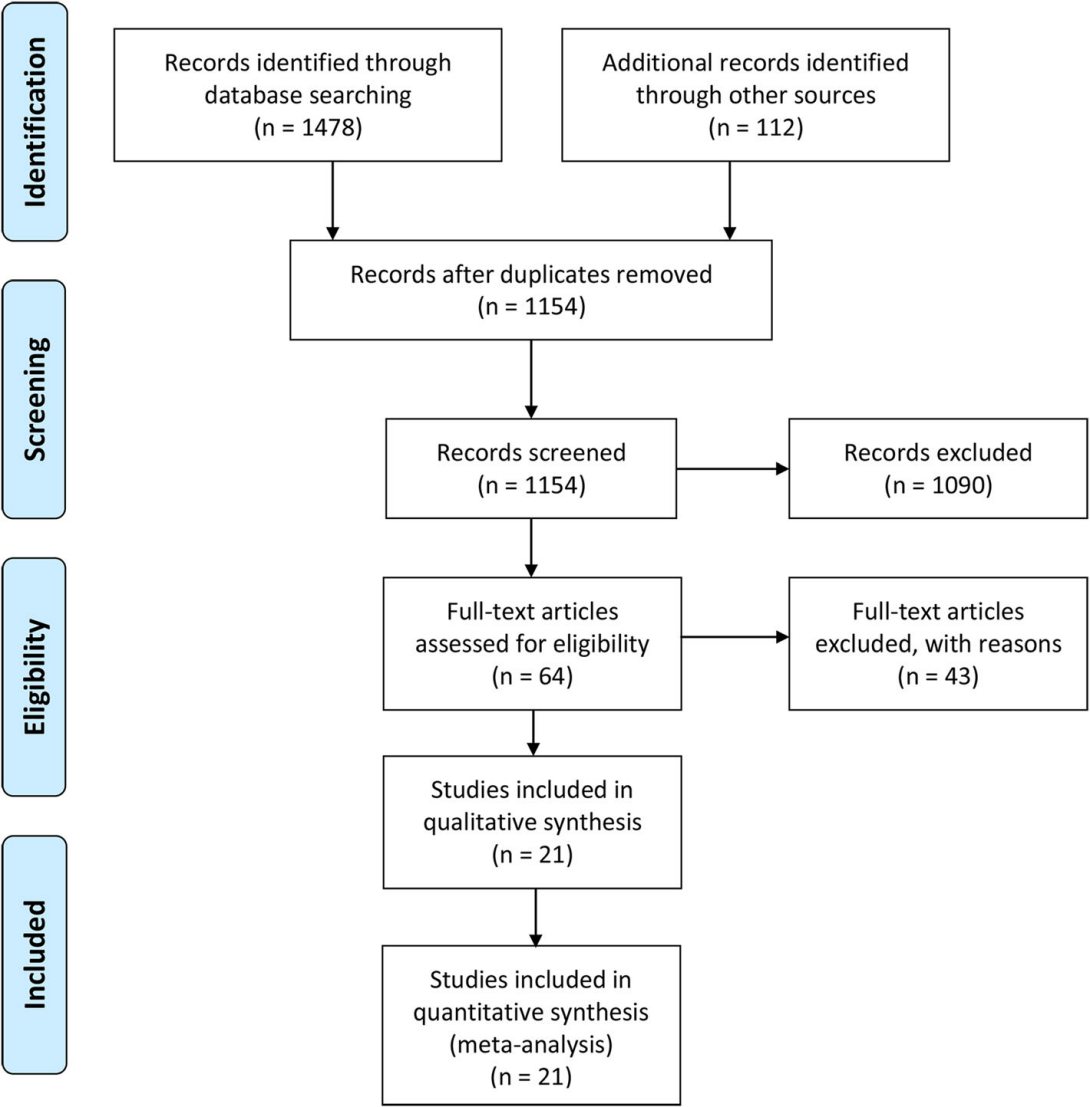
A flowchart of study screening and selection process

**Fig. 2:**
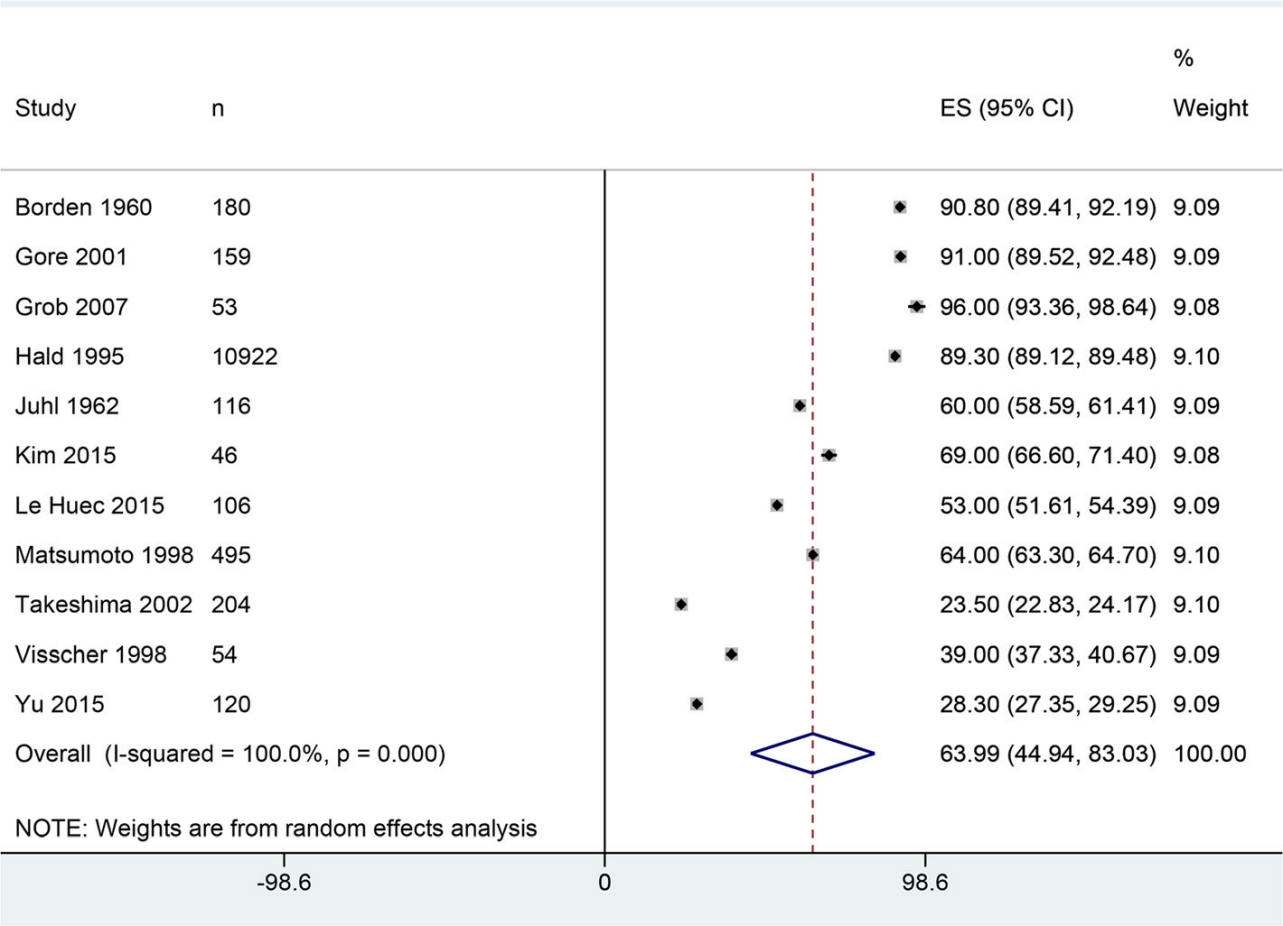
A forest graph showing the percentage of asymptomatic individuals with lordotic cervical curvature and the overall effect size

Twenty-one studies [18, 19, 24–42] were selected by following the eligibility criteria (Fig. 1). In these studies, 15,364 asymptomatic or healthy individuals were recruited. Age (weighted average) of these individuals was 42.30 years [95% confidence interval 36.42, 48.18], and 54.2% were males. Among the asymptomatic individuals studied, 63.99% [44.94, 83.03] had lordotic curvature while the rest had straight, kyphotic, or sigmoid curvature (dataset, 12455 asymptomatic individuals in 11 studies; Fig. 2).

**Fig. 3:**
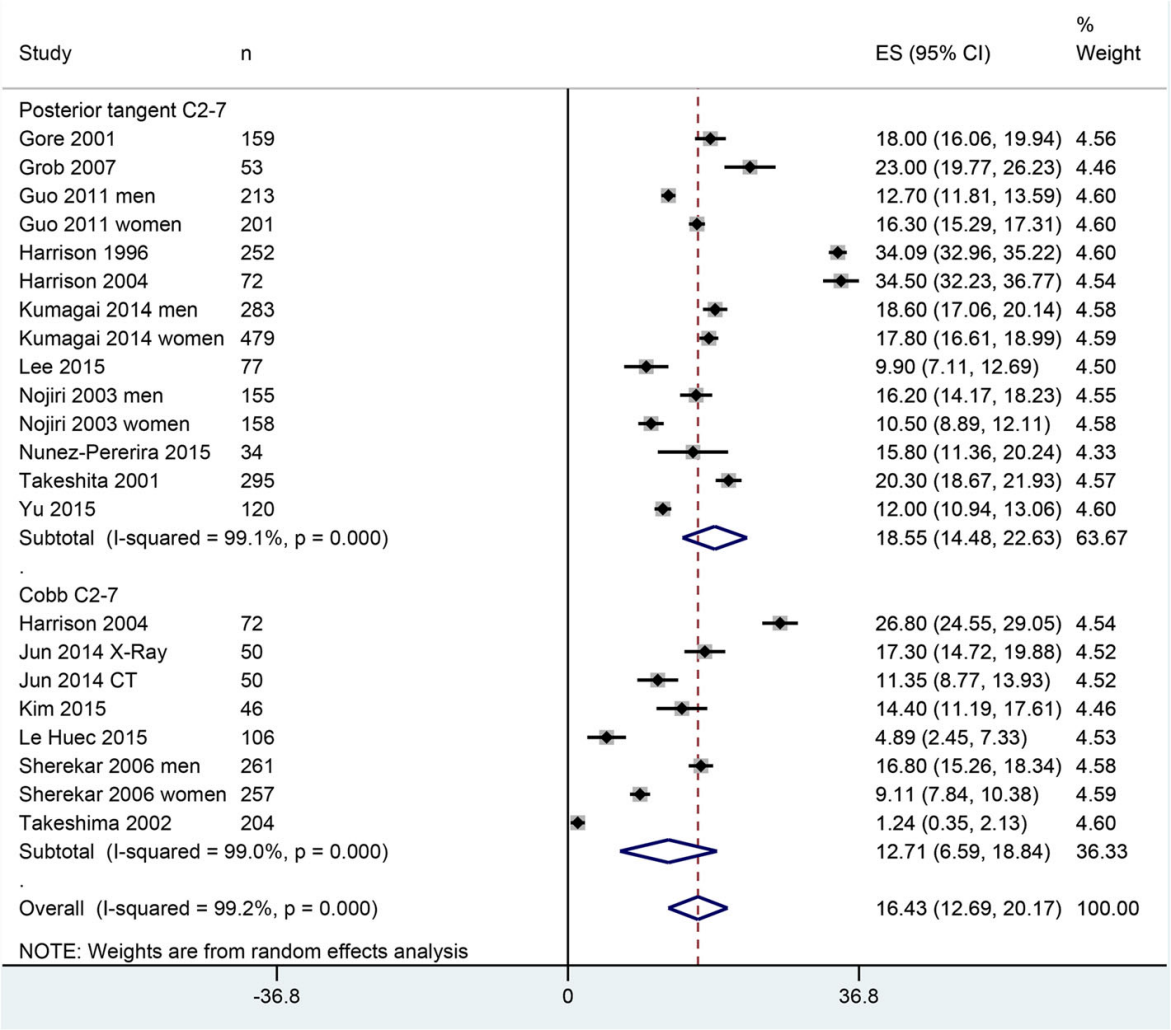
A forest graph showing the cervical lordotic angles reported by individual studies and the overall effect size as well as the effect sizes of two methods used to measure the cervical curvature

Eleven of the included studies reported degree of cervical lordotic curvature in 3597 asymptomatic individuals. Overall, cervical lordotic curvature was found to be 16.43° [95% confidence interval 12.69, 20.17]. However, cervical lordotic curvature differed by the method used. In 1046 asymptomatic individuals who underwent measurements with Cobb C2–C7 method, the curvature was found to be 12.71° [6.59, 18.84], whereas in 2551 asymptomatic individuals who underwent measurements with posterior tangent method, the cervical lordotic curvature was 18.55° [14.48, 22.63] (Fig. 3).

**Fig. 4:**
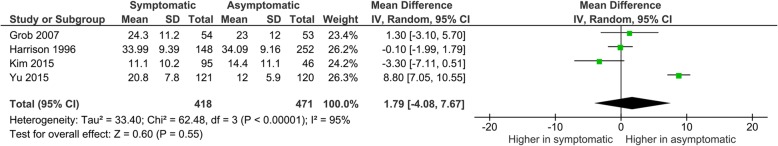
A forest graph showing no significant difference between symptomatic and asymptomatic individuals in the cervical lordotic angles

**Fig. 5:**
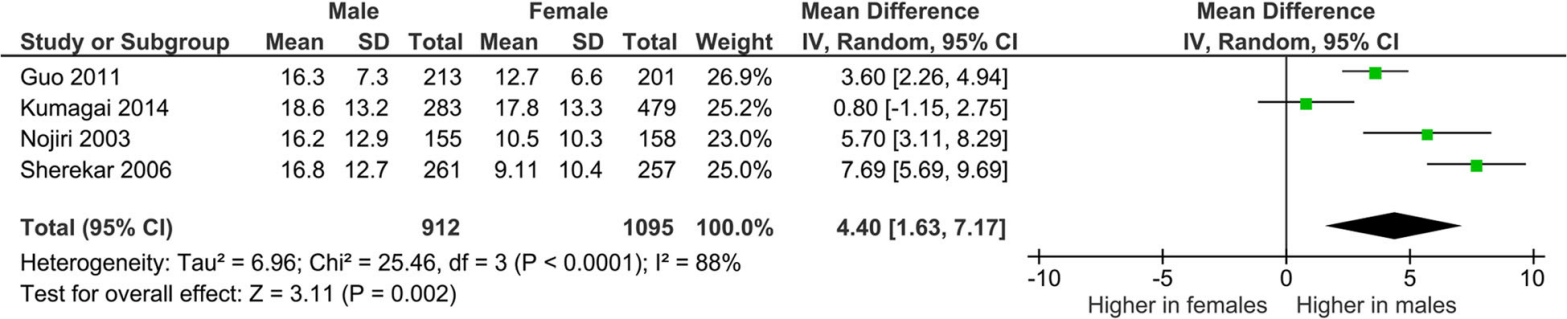
A forest graph showing a significant difference in the cervical lordotic angles between male and female asymptomatic individuals

Lordotic cervical curvature was not significantly different between symptomatic and asymptomatic individuals (mean difference was 1.79° [− 4.08, 7.67]; *p* = 0.55; Fig. 4). However, lordotic cervical curvature was significantly higher in males in comparison with that in females (mean difference 4.4° [1.63, 7.17]; *p* = 0.002; Fig. 5).

**Fig. 6:**
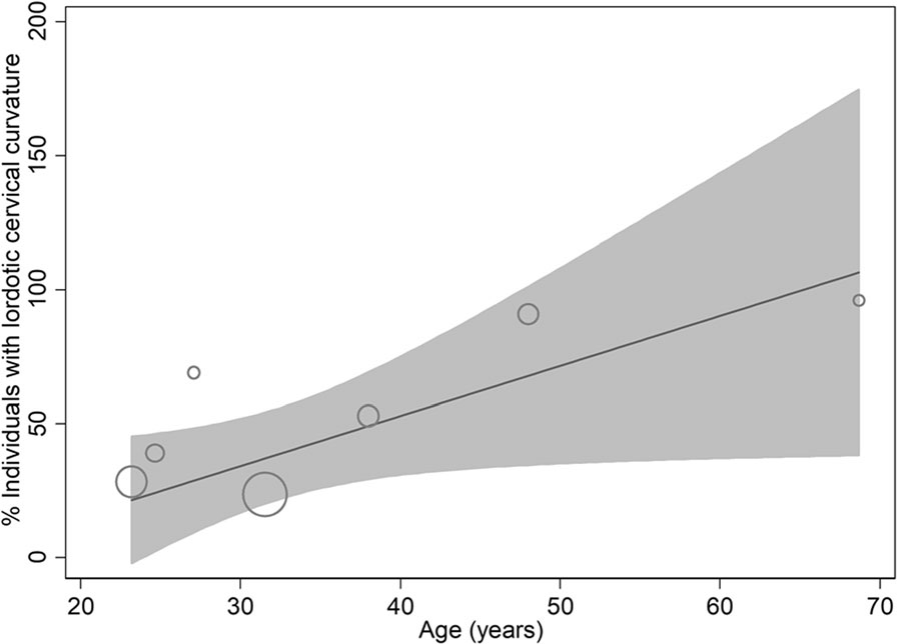
A metaregression scatterplot showing the relationship between age and the percentage of individuals with lordotic curvature in cervical spine

In the meta-regression analyses, age was not significantly associated with lordotic cervical curvature (coefficient 0.18 [− 0.19, 0.56]; *p* = 0.314) but was significantly associated with the percentage of individuals with lordotic cervical curvature (coefficient 1.43 [0.20, 2.66]; *p* = 0.030; Fig. 6).

## Discussion

This meta-analytical review finds that most asymptomatic individuals possess lordotic cervical curvature which averages at about 18° when measured with C2–C7 posterior tangent method. However, Cobb C2–C7 method may underestimate as we have found that use of this method led to an average of about 13° of lordotic cervical angle. We have also observed that there is no significant difference in cervical curvature between symptomatic and asymptomatic individuals but lordotic cervical curvature has been found to be significantly higher in men than in women. However, no significant relationship could be found between lordosis and age, although the percentage of individuals with lordotic cervical curvature increased with age.

In a review study in which the authors analyzed data from studies which evaluated neutral upright sagittal spinal alignment from the occiput to the pelvis in asymptomatic adults, the greatest variation was noted in the cervical spine from C2 to C7 [43]. In this scenario, it becomes difficult to assess the effect of method on cervical curvature, although in a study in which both the methods were used simultaneously, lordotic angle values were found significantly higher with the posterior tangent C2–C7 method than with the Cobb C2–C7 method [19]. A factor that is postulated to affect the level of cervical lordosis is the sagittal shape of odontoid dens [44, 45].

Although meta-regression analysis of the present study could not find a significant association between age and cervical lordotic curvature, increase in lordosis with age is reported by some studies [10, 23]. However, contradictory reports are also available in literature. Milne and Lauder [46], by using an indirect method (surveyor’s flexicurve device) in a cross-sectional study of men and women aged 20–90 years, found an increase in kyphosis with age in both older men and women with no lordosis in an increasingly large proportion of both men and women over 60 years of age. Later, Harrison et al. [15] while comparing measurements with flexicurve and radiographic line methods, found that of the 96 flexicurve detected lordotic individuals, only 55 were found to have lordotic curvature on lateral radiographs. In the present study, we have found a positive relationship between the age and the percentage of individuals with lordotic curvature. However, our sample population belonged to rather a middle-aged group.

Lumbar lordosis, sacral inclination, and lumbosacral angulation show a tendency to decrease in individuals aged over 70 years [47]. Using a non-invasive measuring system in 323 asymptomatic individuals, Dreicharf et al. [48] found that total lordosis was significantly reduced by approximately 20% and the range of motion for maximal upper body flexion (RoF) by 12% and extension (RoE) by 31% in the oldest (50–75 years) compared to those in the youngest age cohort (20–29 years). During aging, the lower lumbar spine retains its lordosis and mobility, whereas the middle part flattens and becomes less mobile [48].

Such changes with age may put pressure on cervical spine to change simultaneously causing changes such as increase in lordosis seen by some authors. Whereas in healthy adults (aged 22–50 years), cervical sagittal alignment is found to be associated with thoracic sagittal alignment but not with lumbopelvic alignment [49] and cervical lordosis inversely correlates with thoracic kyphosis [50]; in elderly people, cervical curvature is affected by pelvic sagittal alignment [51]. Lumbar lordosis decreases, and thoracic kyphosis increases with age which results in a compensatory increase in cervical lordosis [10, 17].

Based on a limited data that could be gained under the eligibility criteria of the present study, there was no significant difference between symptomatic and asymptomatic individuals in lordosis angle of the cervical spine. However, this finding is compatible with several related reports. As reviewed by Lippa et al. [52], many studies have found no association between cervical lordotic curvature and symptoms especially the neck pain including whiplash injury in approximately 700 individuals [53–56]. Exceptions can also be found in literature. McAviney et al. [57], after examining 300 cervical X-rays in individuals with and without cervical pain, found a statistically significant association between cervical pain and lordosis of less than 20° (posterior tangent method). However, in the present study, we have found that majority of the included studies which used the posterior tangent method reported less than 20° cervical lordosis in asymptomatic individuals. Therefore, so far, evidence suggests that there exists no association between the degree of cervical lordosis and related symptoms. Even, there is some evidence to suggest that individuals with kyphotic cervical curvature may remain asymptomatic [54]. One of the weaknesses of the present study is that we could not perform subgroup analysis under hyperlordosis and hypolordosis categories. It was due to unavailability of categorical data in reports of the included studies. Only Harrison et al. described this as “The cervical lordosis in both acute and chronic neck pain patients was found to be hypolordotic.” We did not try to categorize it because of a wide range of lordosis curvature in asymptomatic individuals and different degrees achieved by the use of two main methods in the included studies. Moreover, in general, we found no association between symptoms and lordosis angle.

## Conclusion

Approximately 68% asymptomatic individuals possess lordotic cervical curvature. Average lordotic curvature is estimated at 18° when C2–C7 posterior tangent method was used and 13° with Cobb C2–C7 method. No significant difference in cervical curvature between symptomatic and asymptomatic individuals has been found, but lordotic cervical curvature was significantly higher in men than in women. No significant relationship was found between lordosis and age, but a positive relationship between the age and the percentage of individuals with lordotic curvature is observed.
